# Characterization of Seven Species of *Camellia* Oil: Oil Content, Volatile Compounds, and Oxidative Stability

**DOI:** 10.3390/foods13162610

**Published:** 2024-08-20

**Authors:** Fu-Lan Hsu, Ying-Ju Chen, Chun-Kai Hsu, Liang-Jong Wang

**Affiliations:** 1 Forest Products Utilization Division, Taiwan Forestry Research Institute, Taipei 100051, Taiwan; flhsu@tfri.gov.tw (F.-L.H.); yingju@tfri.gov.tw (Y.-J.C.); 2 Lienhuachih Research Center, Taiwan Forestry Research Institute, Nantou 555002, Taiwan; dakai0327@tfri.gov.tw; 3 Forest Protection Division, Taiwan Forestry Research Institute, Taipei 100051, Taiwan

**Keywords:** *Camellia*, oil content, oil stability index, seed oil, volatile compounds

## Abstract

In this study, we conducted tests on the seeds from four Taiwanese native *Camellia* species (*C. japonica*, *C. furfuracea*, *C. laufoshanensis*, and *C. formosensis*) and three commercialized species (*C. oleifera*, *C. brevistyla*, and *C. sinensis*) for comparison. We examined various aspects of these species, such as seed oil content, suitability for mechanical pressing, volatile components (edible flavor), and oil stability (suitability for cooking), to assess the feasibility of using these four native Taiwanese *Camellia* seeds as sources of edible oil. The results from solvent extraction tests and mechanical pressing experiments confirm that the seeds from *C. furfuracea*, *C. japonica*, and *C. laufoshanensis* have high oil contents, and their oils are suitable for extraction via the popular mechanical pressing method, with oil yields comparable to or higher than those of the commercialized *Camellia* species. The volatile components of the oils were collected using MonoTrap adsorbents and analyzed with a thermal desorption system coupled with gas chromatography–mass spectrometry (ATD-GC/MS), primarily consisting of alcohols, ketones, and aldehydes. The results of oxidative stability tests reveal that the seed oils from *C. japonica*, *C. furfuracea*, and *C. laufoshanensis* are higher than or equally stable to those from the commercialized *Camellia* species. After six months of storage, the stability of these three *Camellia* seed oils remained relatively high, demonstrating that the seed oils from *C. japonica*, *C. furfuracea*, and *C. laufoshanensis* can withstand high temperatures and can be easily preserved for future applications.

## 1. Introduction

**Figure 1:**
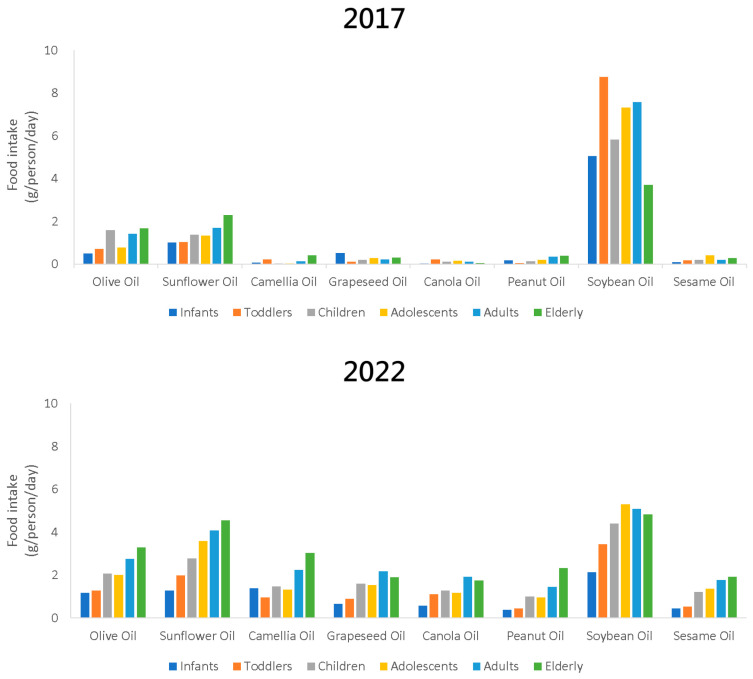
Changes in the dietary habits of the people in Taiwan regarding edible oils (drawn based on [2]).

Many plants are used as oilseeds, and the oils extracted from these plants have diverse applications ranging from high-value functional oils (for medical or healthcare purposes) to skincare, haircare, cosmetics, and aromatherapy base oils, as well as edible cooking oils. These oilseeds can also be applied industrially (ink, paint, waterproofing, coatings, pest control, energy, lighting, etc.), indicating their global value as resources [1]. Among these products, edible oil is one of the most common and highly valued items in the daily lives of the general public. In Taiwan, consumption habits have shifted regarding edible oils [2]. In 2017, the primary oils consumed were soybean, sunflower, and olive oils. This trend has gradually shifted to a wider acceptance of various plant oils, including *Camellia* oil (Figure 1).

In recent years, the global consumption of vegetable oils has substantially increased, owing to particularly the rising demand for mechanically pressed oils [3]. Mechanically pressed oils better retain the natural properties of the oil, including its nutritional value, active components, and unique colors and flavors. Additionally, mechanical pressing is a safe and environmentally friendly extraction method. The process is relatively simple; after pressing, the oil only needs to settle or be filtered and can be consumed without the need for refining. Amidst this trend, interest has increased in exploring new sources of oils. Although less efficient, the oil extracted via mechanical pressing has a rich variety of colors and flavors. Hence, these oils remain popular among consumers despite their relatively high price [3]. Small-to-medium oil mills in Taiwan commonly use physical mechanical pressing methods to extract edible oils from plant seeds. The common physical pressing methods include cake pressing and screw pressing, the latter being more prevalent due to its higher efficiency.

Currently, two types of *Camellia* seed oils are available on the market in Taiwan: Kucha and tea seed oils. Kucha oil contains a high amount of oleic acid and is mainly extracted from the seeds of *C. oleifera*, which has larger fruits, and *C. brevistyla*, which has smaller fruits. Tea seed oil has a lower oleic acid content and is primarily extracted from the seeds of *C. sinensis* [4,5,6,7]. These three types of *Camellia* seed oils are popular and commercialized and have been the subject of numerous studies, including those focused on the optimal harvesting period of the fruits [8]; the active components of the oils (vitamins, phytosterols, triterpenes, lignans, and flavonoid compounds) [9]; the physiological regulatory functions of the oils, such as antioxidation capacity, regulation of blood sugar and lipids, promotion of wound healing, inhibition of tumor growth, and liver protection [10,11,12,13,14]; the effects of seed roasting and cooking oil temperatures on oil properties [15,16,17]; as well as the activity of the cake after oil extraction [18].

**Figure 2:**
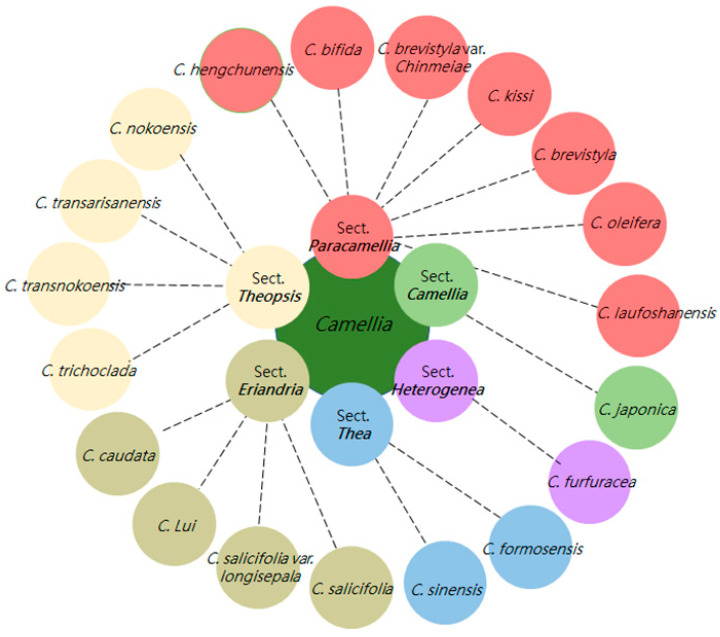
*Camellia* species in Taiwan (drawn based on [19]).

In Taiwan, the genus *Camellia* is treated as 17 taxa (14 species and 3 varieties), assigned to six sections: *Paracamellia*, *Camellia*, *Heterogenea*, *Thea*, *Eriandria*, and *Theopsis* [19], as shown in Figure 2. In addition to the three commercialized *Camellia* seeds, research into other high-seed-yield varieties is urgently needed for the development of distinctive oil products.

Several factors need to be considered when determining whether seeds have the potential to be developed as a source of edible oil: the oil content of the seeds affects the yield, the suitability for mechanical oil extraction determines whether they can be developed into flavorful edible oils, and the stability of the oil influences consumer preferences in terms of cooking options and ease of preservation. Volatile compounds play an important role in the aroma quality of oils. Olive oil is a notable example of high-economic-value oil, for which the analysis of volatile substances is used to identify the unique flavors of oils produced from different varieties, and this result is closely related to consumer preferences [20].

Extensive research has been conducted on the fatty acids, active components, and volatile substances of economically cultivated *Camellia* oils, especially the *C. oleifera*. However, there is a notable lack of data regarding the feasibility of using other potentially valuable non-economically cultivated Taiwanese *Camellia* species—known for their abundant flowering periods and seed yields—as sources of edible oil. Therefore, this study investigates the oil content, mechanical pressing suitability, volatile components related to edible aroma, and stability for cooking of seeds from four native Taiwanese *Camellia* species (*C. japonica*, *C. furfuracea*, *C. laufoshanensis*, and *C. formosensis*) in order to develop novel oil products in the future.

## 2. Materials and Methods

### 2.1. Materials

The fruits of seven *Camellia* species were collected from 3 separate regions in Taiwan (shown in Supplementary Materials (Figure S1)) during the crop season 2022: *C. japonica*, *C. brevistyla*, and *C. oleifera* were from Dongshih District, Taichung City; *C. sinensis* was from Mingjian Township, Nantou County; and *C. furfuracea*, *C. laufoshanensis*, and *C. formosensis* were from Lienhuachih, Yuchi Township, Nantou County. After sun exposure and manual shelling, only seeds without infection or physical damage were placed in an oven at 40 °C for drying until a constant weight was achieved.

### 2.2. Production of Camellia Seed Oils

#### 2.2.1. Solvent Extraction Method

The oil within the seeds was extracted using a Soxhlet extractor using *n*-hexane as the solvent [9]. The solvent was then removed using a vacuum concentrator, and the resulting oil weight (in grams) was obtained after drying. The oil content (%) was calculated by dividing the oil weight by the seed weight and multiplying by 100 [8].

#### 2.2.2. Screw Fresh-Pressing Method

The experimental seeds were processed using a screw press machine (SX-TB05 oil press, Oiling, New Taipei, Taiwan) to extract the seed oil. After the screw-pressing process, the crude oil was centrifuged at 5000 rpm for 15 min. The seeds were not roasted. Finally, the supernatant oil was kept as virgin seed oil in glass vials in a room at 25 °C until analysis. The resulting oil was weighed, and the oil yield was calculated using the following formula [21]:

Oil yield (%) = (weight of oil/weight of seeds) × 100%

The oil was then stored in glass bottles wrapped with aluminum foil to avoid light exposure and preserved in a refrigerator at 4 °C until subsequent analysis.

### 2.3. Volatile Components

#### 2.3.1. VOC Sampling

To collect the components of the oil responsible for flavor, a monolithic silica adsorbent for thermal desorption (MonoTrap RGPS TD, GL Sciences Inc., Tokyo, Japan) was placed at fixed positions in the headspace of a 40 mL glass vial containing approximately 4 mL of an oil sample, and a septum was hermetically sealed on the vial. The vial was maintained in a 40 °C water bath for 5 h to collect flavor components. Then, the MonoTrap adsorbent containing the collected flavor components was placed in a glass tube specialized for thermal desorption systems (MonoTrap TD Liner for OPTIC/LINEX, GL Sciences Inc., Tokyo, Japan) and stored at −20 °C until analysis.

#### 2.3.2. ATD-GC/MS Analysis

Headspace volatiles that were collected using the MonoTrap were analyzed by gas chromatography–mass spectrometry (GC-MS) (Clarus 600 GC-MS system, PerkinElmer Instruments, Waltham, MA, USA) equipped with a thermal desorption system (Turbo Matrix ATD, PerkinElmer Instruments, Waltham, MA USA). Before desorption, sample tubes were purged with pure nitrogen for 1 min at ambient temperature to remove excess humidity, and a fixed amount of gaseous internal standard chlorobenzene d5 (Sigma-Aldrich, Taufkirchen, Germany; Product#: 48086, CAS#: 3114-55-4) was automatically introduced onto the sample tube for internal standard calibration. During the first desorption step, sampling tubes were desorbed at 240 °C for 20 min at 30 mL/min onto a Tenax^®^ TA 60/80-packed cold trap (PerkinElmer) at −30 °C. The second desorption involved heating at a rate of 40 °C/s to a final temperature of 280 °C for 12 min. The terpenoids were separated using a fused silica capillary column (DB-5ms, length of 30 m, i.d. of 0.25 mm, film of 0.25 μm) (Agilent Technologies, Taipei, Taiwan). The initial oven temperature was 35 °C; the samples were held in the oven at this temperature for 5 min, and then subjected to the following program: from 35 to 60 °C at 0.4 °C/min, to 100 °C at 3 °C/min, to 260 °C at 35 °C/min, and maintained at 260 °C for 5 min. Helium was the carrier gas, which was used at a constant flow of 1 mL/min. The temperatures of the GC injector and transfer line were both 250 °C. The MS detector was set up to 230 °C in the scan mode with the *m*/*z* ranging from 15 to 350 amu.

The compounds were tentatively identified by comparing the mass spectra and arithmetic index (AI) with the mass spectra library and the reference AI (rAI) [22]. The AIs were calculated for all volatile constituents by using a homologous series of *n*-alkanes (C_8_–C_23_) on the DB-5ms column. The MS databases that we used included the Wiley/NBS Registry of Mass Spectral Database (version 7) and NIST MS Search (version 2). The relative amounts of each component were calculated based on the integrated peak areas of the chromatograms, and the data are presented as the mean ± standard deviation of three replicates. Cluster analyses were performed with MVSP (multi-variate statistical package for Windows ver. 3.1., Kovach Computing Services) [23] to evaluate the similarity of the volatile constituents of the *Camellia* seed oils.

### 2.4. Oxidative Stability Analysis

Following CNS 14876 (2004) standards [24] and Chan et al. [21], the oil stability index (OSI) analysis was conducted using an oil oxidative stability analyzer (892 Professional Rancimat, Metrohm, Herissau, Switzerland). We weighed 5 g of oil, which was placed in an environment with an air flow rate of 9 L/h at a temperature of 120 °C. The analyzer was used to measure the volatile substances produced by the oil under high-temperature and ventilated conditions, which are soluble in water and cause a change in the conductivity of the water. Our study aimed to understand the effect of room temperature storage on the oxidative stability of the oil. Therefore, the freshly pressed oil was stored in glass bottles and kept in a dark, room temperature environment for six months until conducting oxidative stability analysis. OSI is the point at which the conductivity sharply increases during the test, indicating the time at which large amounts of degradation products are produced in the oil.

### 2.5. Statistical Analyses

The results are expressed as the mean ± standard error (*n* = 3). The significance of differences among individual means was assessed using Scheffe’s multiple comparison procedure in SPSS program package (Statistical Product and Service Solutions, Version 24.0). Differences with *p* < 0.05 were considered statistically significant.

## 3. Results and Discussion

**Figure 3:**
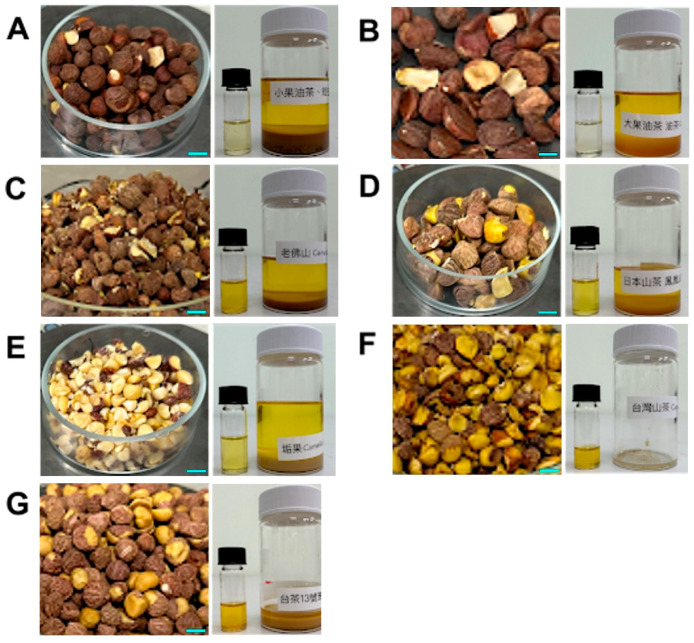
Appearance of experimental seeds and the corresponding oils extracted (small bottles: solvent-extracted oil; large bottles: screw-pressed oil). (**A**) *Camellia brevistyla*; (**B**) *Camellia oleifera*; (**C**) *Camellia laufoshanensis*; (**D**) *Camellia japonica*; (**E**) *Camellia furfuracea*; (**F**) *Camellia formosensis*; (**G**) *Camellia sinensis* cv. TTES No.13. (Scale bar = 1 cm).

The appearance of the experimental seeds and the corresponding extracted oils is shown in Figure 3. The seeds of *C. oleifera* are the largest, followed by those of *C. japonica* and *C. brevistyla*, among the four native *Camellia* oils in Taiwan.

### 3.1. Seed Oil Content

The solvent extraction method efficiently extracts oil; so, the amount of oil obtained via solvent extraction can be considered as the oil content of the seeds. The oil contents of the tested seeds shown in Table 1 ranges from 24.3% to 59.7%, which, arranged from the highest to the lowest, were as follows: *C. furfuracea* (59.7%), *C. japonica* (54.1%), *C. laufoshanensis* (50.3%), and *C. formosensis* (24.3%). The oil contents of the three commercialized *Camellia* species seeds were 44.5% for *C. brevistyla*, 53.3% for *C. oleifera*, and 32.0% for *C. sinensis*. These results are highly consistent with the findings of Robards et al. [25], which reported that the oil content of traditional *Camellia* species seeds ranges from 24% to 50%, with an average of 30%. The oil contents of *C. furfuracea*, *C. japonica*, and *C. laufoshanensis* are higher than or equivalent to those of the commercialized species and the results of a previous study [25], indicating their suitability for future development and application. The lower oil contents of *C. formosensis* and *C. sinensis*, both belonging to Sect. Thea, align them within the same taxonomic group.

### 3.2. Suitability for Mechanical Oil Pressing

In order to understand the characteristics of oils produced through physical mechanical pressing, oil was extracted from the seeds of seven native Taiwanese *Camellia* species using a screw press machine. The oil yields are shown in Table 1. Among the four native *Camellia* species, the highest oil yield was from *C. furfuracea* (49.4%), followed by *C. japonica* (45.7%) and *C. laufoshanensis* (32.5%), with the lowest yield from *C. formosensis* (1.2%). The oil yields of the three commercialized *Camellia* species (*C. brevistyla*, *C. oleifera*, and *C. sinensis*) were 32.3%, 37.1%, and 8.7%, respectively. These results of the three commercialized *Camellia* oils are similar to those reported in a previous study [3], which found that the oil yields from pressing the seeds of *C. brevistyla*, *C. oleifera*, and *C. sinensis* were 35.6%, 33.6%, and 12.4%, respectively. The oil yields of *C. furfuracea*, *C. japonica*, and *C. laufoshanensis* are higher than or equivalent to those of the commercialized species and the results [3], indicating that these three seed types have a high oil content, and their seeds are suitable for the two popular mechanical pressing methods. However, *C. formosensis* has a low oil content and shows its oil yield is even lower oil when subjected to mechanical pressing, suggesting that both *C. formosensis* and *C. sinensis* are less appropriate for use under the applied screw-pressing conditions. The optimization of oil extraction conditions is still needed to increase the oil yield from the seeds of these two species.

In comparing the oil yields from four native Taiwanese *Camellia* species seeds using the solvent extraction and screw-pressing methods, we found that the trends were consistent: solvent extraction resulted in higher oil yields. This result may be related to the residual oil remaining in the cake after screw pressing. The oil yields from screw pressing were particularly low for *C. sinensis* and *C. formosensis*, indicating that these two types of seed oils are less efficiently extracted under the same screw-pressing conditions.

### 3.3. Volatile Components

**Figure 4:**
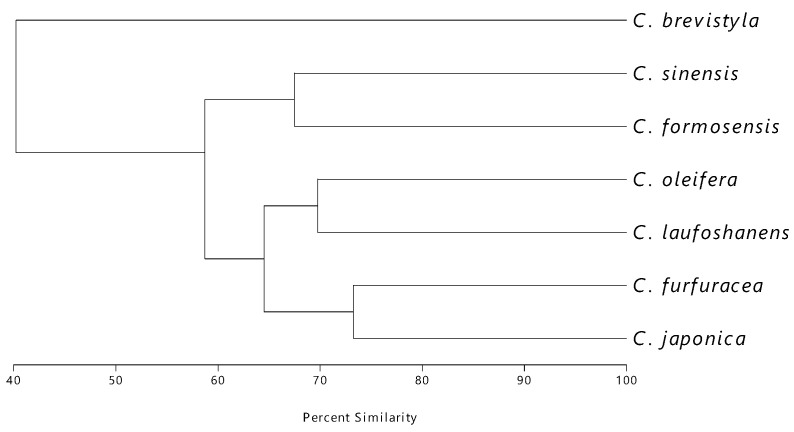
Cluster analysis of different *Camellia* seed oils for the comparison of the similarity in volatile chemical composition.

The flavor properties of an oil are directly correlated with its value for the consumer and determine the success or failure of the product on the market. As such, the analysis of the volatile components of an oil can provide an understanding of this volatile characteristics. The results of the analysis of the volatile components of the different *Camellia* seed oils are shown in Table 2, which primarily consist of alcohols (35.4–51.8%), ketones (6.8–33.2%), and aldehydes (3.0–48.7%) (chromatogram is presented in Supplementary Materials (Figure S2)). The results of cluster analysis (Figure 4) reveal distinct groupings among the *Camellia* species. *C. brevistyla* formed an isolated cluster with a low flavor similarity of only 40.2% compared to the other species. In contrast, *C. oleifera*, *C. laufoshanensis*, *C. furfuracea*, and *C. japonica* formed a separate cluster with a higher degree of flavor similarity (64.5%), indicating a closer relationship among these four species. Within this cluster, *C. oleifera* and *C. laufoshanensis* exhibited a higher similarity (69.8%), as did *C. furfuracea* and *C. japonica* (73.2%). Additionally, *C. sinensis* and *C. formosensis* formed another distinct cluster, with a flavor similarity of 67.5% between them. The main volatile components in *C. brevistyla* seed oil are hexanal (33.9%), an aldehyde, and alcohols, such as 1-pentanol (17.0%), 1-hexanol (8.5%), and isopentyl alcohol (6.2%). Hexanal has a green, leafy, and woody aroma. It can be a naturally occurring component or the main decomposition product of linoleic acid-13-COOH, which is produced via β-homogenous cracking [26]. Oils with hexanal as their primary component have a pronounced fresh fruit and plant aroma, adding a fresh flavor to food [27]. *C. sinensis* and *C. formosensis* are classified into the same group (Figure 4), with a chemical similarity of 67.67%; they are primarily characterized by high levels of the ketone compound acetoin (13.7–28.4%) and the alcohol compound isopentyl alcohol (9.5–20.0%). These ketone and alcohol compounds are typically associated with sweet and creamy aromas; so, these two oils have a similar flavor profile, with *C. formosensis* having a higher proportion of the aldehyde hexanal (11.5%). Additionally, *C. furfuracea*, *C. japonica*, *C. laufoshanensis*, and *C. oleifera* are grouped together with a similarity of 64.64%, with *C. furfuracea* and *C. japonica* (similarity 73.27%), and *C. laufoshanensis* and *C. oleifera* (similarity 69.83%) having closely related chemical components. *C. furfuracea* and *C. japonica* are primarily characterized by the alcohol compounds [R-(R*,R*)]-2,3-butanediol (12.2% and 14.0%, respectively), isopentyl alcohol (10.4% and 15.8%, respectively), and 2,3-butanediol (9.1% and 9.2%, respectively), as well as the ketone compound butyrolactone (19.1% and 19.2%, respectively). Both have high levels of isopentyl alcohol, which imparts a fruity aroma and sweet taste [28], whereas *C. japonica* contains a higher proportion of 2,5-dimethyl-pyrazine (12.0%), a nitrogen-containing pyrazine compound that typically imparts roasted, nutty, and earthy flavor characteristics to foods [29]. Finally, *C. oleifera* and *C. laufoshanensis* contain high proportions of butyrolactone (14.1% and 13.2%, respectively) and isopentyl alcohol (9.3% and 13.5%, respectively). The ketone compound butyrolactone usually has creamy and caramel-like flavor characteristics [30], providing *C. oleifera* and *C. laufoshanensis* with a mild creamy aroma. Hexanal (11.6%, 13.7%) also imparts a fruity and fresh plant aroma. The content of 2,5-dimethyl-pyrazine in *C. oleifera* is higher (18.7%), enhancing its nutty, toasted bitter flavor. The genus *Camellia* is classified based on its morphological characteristics [31], and the sectional level of the seven *Camellia* species in this study is shown in Figure 2. The results of the cluster analysis of the volatile components in different *Camellia* seed oils are consistent with many other molecular phylogenetic findings [32,33,34] but inconsistent with the morphological classification [31].

### 3.4. Oil Stability

During processing and storage, oils are prone to oxidation due to exposure to environmental factors such as oxygen, heat, and light, which can degrade their quality. Therefore, oxidative stability is a key characteristic in evaluating the shelf life and suitability of oils. Conducting oxidative stability analysis at room temperature is a time-consuming process; hence, we employed the Rancimat method to assess the oxidative stability of the oils [35,36,37]. Oxidative stability tests were not conducted on *C. formosensis* seed oil because of the low yield of the oil obtained from the mechanical pressing of *C. formosensis* seeds. The oxidative stability results of the different oils are shown in Table 3, which indicate that the seed oil from *C. japonica* has the highest OSI, followed by those of *C. furfuracea*, *C. oleifera*, *C. laufoshanensis*, and *C. sinensis*. Because the oxidative stability of the native *Camellia* seed oils from *C. japonica*, *C. furfuracea*, and *C. laufoshanensis* is higher than or comparable to that of the three commercially available *Camellia* seed oils and the results reported by Zeng et al. [38], these native *Camellia* seed oils have the potential for high-temperature applications and storage as oils with high oxidative stability, which offer wider versatility in cooking, allowing for various culinary applications such as in salads, stir-frying, pan-frying, and deep-frying. As the storage duration increases, the quality of oils gradually deteriorates due to oxidation. After six months of storage, the oxidative stability of all seven *Camellia* seed oils decreased; however, the OSI of the seed oils from *C. japonica* and *C. furfuracea* remained higher than that of the three commercially available *Camellia* seed oils before storage, demonstrating the oxidative stability of these native *Camellia* seed oils. We also found that *C. brevistyla* seed oil, having the lowest OSI (0 month 0.8 h; 6 months 0.5 h), contains the highest level of hexanal in the volatile component analysis. Hexanal was proposed as an oxidative marker in oil after high-temperature storage [39]. This might indicate that, in addition to fatty acid composition, antioxidant content, levels of free fatty acids, and peroxide content [20], the relative content of hexanal can be used to assess the oxidative stability of oils.

## 4. Conclusions

We evaluated the potential of native Taiwanese *Camellia* seeds to be developed as edible oils from four perspectives: oil content, suitability for mechanical pressing, volatile components, and oil stability. The results show that, among the four native Taiwanese *Camellia* species known for their abundant seed production, the seeds of *C. furfuracea*, *C. japonica*, and *C. laufoshanensis* have a high oil content and are suitable for oil extraction via mechanical pressing. The obtained oils exhibited oxidative stability, even surpassing that of the commercial species. The oils obtained from different *Camellia* species have unique flavors, which are beneficial for future product development.

## Figures and Tables

**Table 1:** Oil content (%) of *Camellia* seeds extracted via solvent extraction and screw-pressing method.

Oil Content (%)							
	Taiwan Native *Camellia* spp.				Taiwan Commercial *Camellia* spp.		
Sect.	*Camellia*	*Heterogenea*	*Paracamellia*	*Thea*	*Paracamellia*		*Thea*
Species	*C. japonica*	*C. furfuracea*	*C. laufoshanensis*	*C. formosensis*	*C. brevistyla*	*C. oleifera*	*C. sinensis*
Solvent extraction	54.1	59.7	50.3	24.3	44.5	53.3	32.0
Screw pressing	45.7	49.4	32.5	1.2	32.3	37.1	8.7

**Table 2:** Volatile constituents of *Camellia* seed oils.

Compounds	AI #	Area (%) ##						
		*C. japonica*	*C. furfuracea*	*C. laufo-* *shanensis*	*C. formosensis*	*C. brevistyla*	*C. oleifera*	*C. sinensis*
2-Pentanone	689	0.2 ± 0.2 ^cd^	1.0 ± 0.2 ^ab^	0.5 ± 0.1 ^bc^	0.1 ± 0.1 ^d^	1.2 ± 0.1 ^a^	0.1 ± 0.0 ^cd^	1.1 ± 0.2 ^a^
Pentanal	695	0.2 ± 0.2 ^b^	1.0 ± 0.2 ^b^	1.6 ± 0.4 ^b^	5.3 ± 2.6 ^a^	2.9 ± 0.7 ^ab^	1.0 ± 0.2 ^b^	1.9 ± 0.2 ^ab^
Acetoin	700	6.8 ± 1.5 ^c^	6.9 ± 0.5 ^c^	3.0 ± 1.8 ^cd^	13.7 ± 3.7 ^b^	0.5 ± 0 ^d^	3.3 ± 0.8 ^cd^	28.4 ± 0.1 ^a^
3-Methyl-butanenitrile	718	4.2 ± 1.4 ^ab^	5.7 ± 1.6 ^a^	1.6 ± 0.4 ^b^	3.7 ± 1.1 ^ab^	1.6 ± 0.2 ^b^	1.4 ± 0.2 ^b^	4.3 ± 1.2 ^ab^
Isopentyl alcohol	723	15.8 ± 4.8 ^ab^	10.4 ± 1.8 ^bc^	13.5 ± 3.5 ^abc^	9.5 ± 1.3 ^bc^	6.2 ± 0.7 ^c^	9.3 ± 2.0 ^bc^	20.0 ± 2.9 ^a^
2-Methyl-1-Butanol	725	4.3 ± 1.8 ^b^	4.6 ± 1.4 ^b^	10.7 ± 2.7 ^a^	2.2 ± 0.2 ^b^	1.6 ± 0.4 ^b^	1.7 ± 0.7 ^b^	2.6 ± 2.3 ^b^
1-Pentanol	754	0.8 ± 0.1 ^c^	1.4 ± 0.2 ^c^	3.9 ± 0.7 ^b^	4.2 ± 0.2 ^b^	17.0 ± 1.3 ^a^	4.9 ± 1.3 ^b^	4.9 ± 0.3 ^b^
2,3-Butanediol	774	9.2 ± 2.2 ^ab^	9.1 ± 1.7 ^ab^	7.8 ± 2.1 ^bc^	15.6 ± 3.6 ^a^	1.7 ± 0.7 ^c^	10.4 ± 1.6 ^ab^	7.6 ± 1.6 ^bc^
[*R*-(R*,R*)]-2,3-Butanediol	788	14.0 ± 4.6 ^a^	12.2 ± 2.5 ^a^	8.9 ± 3.7 ^ab^	13.3 ± 3.3 ^a^	1.7 ± 0.8 ^b^	5.8 ± 1.2 ^ab^	7.2 ± 2.3 ^ab^
Hexanal	796	1.5 ± 0.2 ^c^	9.1 ± 1.8 ^bc^	13.7 ± 2.7 ^b^	11.5 ± 3.3 ^b^	33.9 ± 5.6 ^a^	11.6 ± 0.9 ^b^	8.9 ± 0.5 ^bc^
Methyl-pyrazine	813	2.5 ± 0.3 ^b^	0.2 ± 0.2 ^c^	n.d.	0.3 ± 0.1 ^c^	n.d.	5.1 ± 0.4 ^a^	0.2 ± 0.0 ^c^
2-Methylbutanoic acid ethyl ester	840	0.8 ± 0.1 ^b^	1.2 ± 0.3 ^a^	0.4 ± 0.1 ^bc^	0.1 ± 0.1 ^c^	n.d.	0.1 ± 0.0 ^c^	0.4 ± 0.1 ^bc^
1-Methoxy-2-propyl acetate	861	0.9 ± 0.8 ^ab^	4.1 ± 2.7 ^a^	n.d.	3.5 ± 0.1 ^ab^	n.d.	n.d.	0.5 ± 0.8 ^ab^
1-Hexanol	863	0.4 ± 0.5 ^b^	0.7 ± 1.2 ^b^	5.7 ± 0.8 ^a^	n.d.	8.5 ± 1.7 ^a^	2.2 ± 0.2 ^b^	1.6 ± 0.3 ^b^
Heptanal	901	0.1 ± 0.1 ^b^	0.5 ± 0.4 ^b^	1.6 ± 0.5 ^a^	0.6 ± 0.2 ^b^	2.0 ± 0 ^a^	0.6 ± 0.2 ^b^	0.5 ± 0.2 ^b^
Butyrolactone	903	19.2 ± 3.1 ^a^	19.1 ± 3.9 ^a^	13.2 ± 2.8 ^ab^	2.7 ± 0.8 ^c^	4.4 ± 0.3 ^bc^	14.1 ± 4.5 ^a^	3.7 ± 0.7 ^c^
2,5-Dimethyl-pyrazine	906	12.0 ± 1.3 ^b^	0.1 ± 0.1 ^c^	0.2 ± 0.4 ^c^	1.1 ± 0.3 ^c^	n.d.	18.7 ± 2.1 ^a^	0.4 ± 0.2 ^c^
Benzaldehyde	946	0.2 ± 0.0 ^d^	1.1 ± 0.1 ^ab^	0.8 ± 0.1 ^bc^	1.3 ± 0.4 ^a^	0.5 ± 0.1 ^bcd^	0.5 ± 0.0 ^bcd^	0.3 ± 0.2 ^cd^
(*E*)-2-Heptenal	947	n.d.	0.0 ± 0.1 ^c^	n.d.	1.3 ± 0.2 ^a^	0.7 ± 0.1 ^b^	0.4 ± 0.1 ^bc^	0.4 ± 0.2 ^bc^
1-Heptanol	966	n.d.	0.1 ± 0.1 ^b^	0.9 ± 0.3 ^b^	0.1 ± 0.1 ^b^	2.5 ± 0.8 ^a^	0.9 ± 0.2 ^b^	0.1 ± 0.1 ^b^
Octanal	1002	0.0 ± 0.1 ^b^	0.7 ± 0.2 ^b^	3.6 ± 0.7 ^a^	0.5 ± 0.1 ^b^	4.6 ± 1.2 ^a^	1.6 ± 0.3 ^b^	0.2 ± 0.1 ^b^
Benzeneacetaldehyde	1031	n.d.	1.3 ± 0.6 ^a^	0.0 ± 0.1 ^b^	n.d.	0.1 ± 0.1 ^b^	0.4 ± 0.2 ^b^	n.d.
Nonanal	1104	0.2 ± 0.2 ^b^	3.3 ± 1.2 ^a^	3.1 ± 1.1 ^a^	1.3 ± 0.2 ^ab^	2.6 ± 0.8 ^ab^	1.3 ± 0.4 ^ab^	0.4 ± 0.3 ^b^
*trans*-Methyl cinnamate	1382	2.7 ± 1.7 ^a^	n.d.	0.1 ± 0.0 ^b^	n.d.	n.d.	0.1 ± 0.0 ^b^	n.d.
(*E*,*E*)-α-Farnesene	1505	n.d.	n.d.	n.d.	2.3 ± 0.2 ^a^	n.d.	n.d.	0.3 ± 0.1 ^b^
1-Heptadecene	1694	0.2 ± 0.1 ^a^	1.1 ± 0.8 ^a^	0.3 ± 0.1 ^a^	0.6 ± 0.5 ^a^	0.2 ± 0.1 ^a^	0.2 ± 0.1 ^a^	0.3 ± 0.1 ^a^
Ketones		26.2 ± 2.5 ^abc^	27.2 ± 3.5 ^ab^	17.1 ± 3.4 ^c^	16.7 ± 3.3 ^c^	6.8 ± 0.3 ^d^	17.8 ± 4 ^bc^	33.2 ± 0.6 ^a^
Aldehydes		3.0 ± 0.9 ^d^	19.8 ± 3.1 ^bc^	25.5 ± 1.4 ^b^	23.8 ± 7.4 ^bc^	48.6 ± 3.8 ^a^	18.7 ± 0.7 ^bc^	13.6 ± 0.4 ^cd^
Nitrogen-containing compounds		18.8 ± 0.4 ^b^	6.0 ± 1.7 ^c^	2.4 ± 0.1 ^cd^	5.2 ± 0.7 ^cd^	1.6 ± 0.2 ^d^	25.3 ± 2.5 ^a^	5.0 ± 1.1 ^cd^
Alcohols		44.6 ± 0.8 ^abc^	39.0 ± 2.1 ^bc^	51.8 ± 2.5 ^a^	45.4 ± 5.6 ^ab^	40.5 ± 3.1 ^bc^	35.4 ± 0.7 ^c^	44.1 ± 1 ^abc^
Furanic compounds		1.3 ± 0.0 ^ab^	0.6 ± 0.1 ^d^	1.2 ± 0.3 ^abc^	1.0 ± 0.2 ^abcd^	0.6 ± 0.2 ^cd^	1.6 ± 0.1 ^a^	0.8 ± 0.2 ^bcd^
Esters		6.0 ± 1.6 ^a^	6.2 ± 2.7 ^a^	1.6 ± 0.4 ^bc^	4.7 ± 0.6 ^ab^	0.2 ± 0.2 ^c^	0.7 ± 0.1 ^bc^	1.0 ± 1.0 ^bc^
Terpenes		0.0 ± 0.0 ^c^	0.0 ± 0.0 ^c^	0.0 ± 0.0 ^c^	2.3 ± 0.2 ^a^	0.0 ± 0.0 ^c^	0.0 ± 0.0 ^c^	0.3 ± 0.1 ^b^

**#** AIs relative to *n*-alkanes (C_8_–C_23_) on a DB-5 ms column; **##** Only components with relative abundance > 1% are listed in this table; Different letters in the table denote significant differences among the species (*p* < 0.05).

**Table 3:** Oil stability indices (h) of *Camellia* oils.

	Taiwan Native *Camellia* spp.			Taiwan Commercial *Camellia* spp.		
Sect.	*Camellia*	*Heterogenea*	*Paracamellia*	*Paracamellia*		*Thea*
Species	*C. japonca*	*C. furfuracea*	*C. laufoshanensis*	*C. brevistyla*	*C. oleifera*	*C. sinensis*
0 month	10.4 ± 0.0 ^a^	8.4 ± 0.0 ^b^	3.8 ± 0.0 ^d^	0.8 ± 0.1 ^f^	4.3 ± 0.0 ^c^	1.5 ± 0.0 ^e^
6 months	7.5 ± 0.0 ^b^	8.1 ± 0.0 ^a^	1.6 ± 0.0 ^c^	0.5 ± 0.0 ^e^	1.4 ± 0.1 ^cd^	1.4 ± 0.0 ^d^

Different letters in the table denote significant differences among the species (*p* < 0.05).

## Data Availability

The original contributions presented in the study are included in the article and Supplementary Materials, further inquiries could be directed to the corresponding author.

## Supplementary Materials

The following supporting information can be downloaded at: https://www.mdpi.com/article/10.3390/foods13162610/s1, Figure S1: Map of the collecting sites of seven Camellia species. Figure S2: GC/MS chromatogram of volatile compounds of seed oil from Camellia species.
