# Supplementary material for: Characterization of Seven Species of Camellia Oil: Oil Content, Volatile Compounds, and Oxidative Stability
Source: Foods. 2024 Aug 20;13(16):2610. doi: 10.3390/foods13162610 (PMC11353628; doi:10.3390/foods13162610)
Supplement: Supplementary file 1 [file foods-13-02610-s001.zip › foods-3124222-supplementary.pdf]

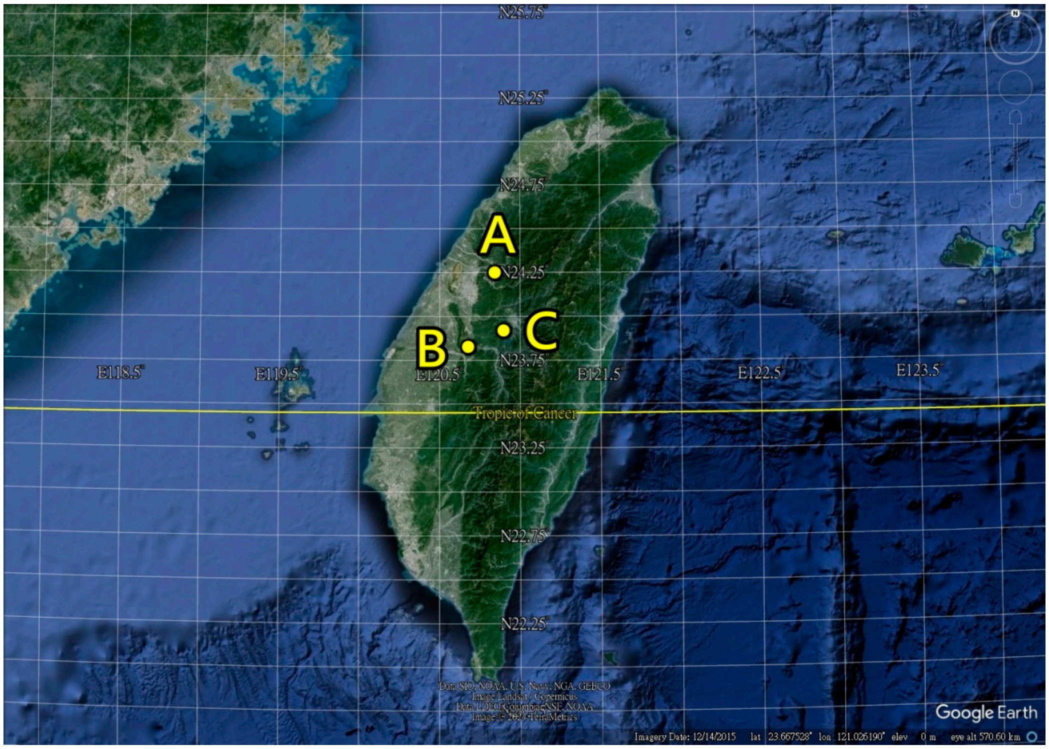

Figure S1. Map of the collecting sites of seven *Camellia* species. A. Dongshih District, Taichung City; B. Mingjian Township, Nantou County; C. Lienhuachih, Yuchi Township, Nantou County.

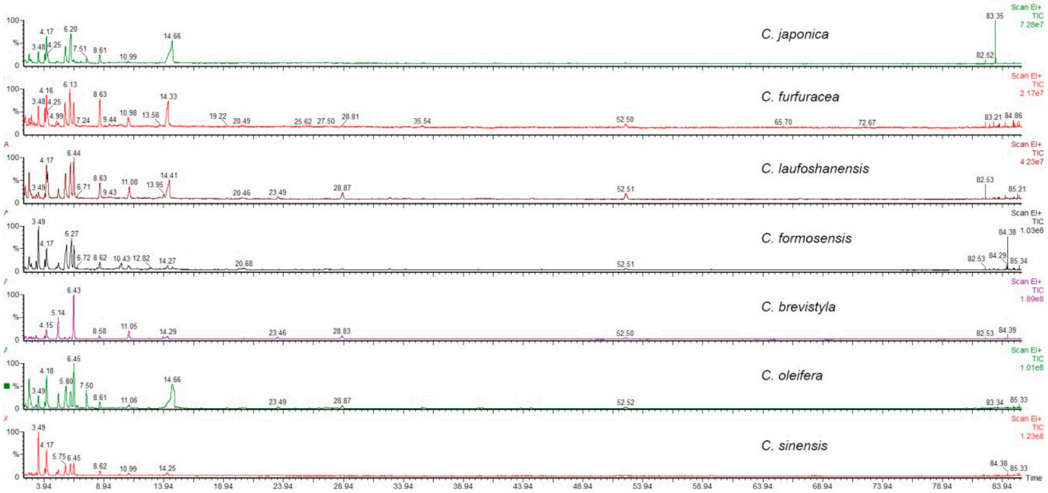

Figure S2. GC/MS chromatogram of volatile compounds of seed oil from *Camellia* species.
